# Genotypic Characterization of Epstein Barr Virus in Blood of Patients with Suspected Nasopharyngeal Carcinoma in Ghana

**DOI:** 10.3390/v12070766

**Published:** 2020-07-16

**Authors:** Richmond Ayee, Maame Ekua Oforiwaa Ofori, Emmanuel Ayitey Tagoe, Sylvester Languon, Kafui Searyoh, Louis Armooh, Estella Bilson-Amoah, Kenneth Baidoo, Emmanuel Kitcher, Edward Wright, Osbourne Quaye

**Affiliations:** 1 West African Center for Cell Biology of Infectious Pathogens (WACCBIP), Department of Biochemistry, Cell and Molecular Biology, University of Ghana, Legon, Accra 00233, Ghana; ayeerichmond@gmail.com (R.A.); oofori15@gmail.com (M.E.O.O.); eatagoe@ug.edu.gh (E.A.T.); slsleuthy@gmail.com (S.L.); 2 Ear, Nose and Throat Unit, Korle-Bu Teaching Hospital, Accra 00233, Ghana; kksearyoh@gmail.com (K.S.); larmooh@yahoo.com (L.A.); ebilsonamoah@gmail.com (E.B.-A.); dr3kbaidoo@yahoo.com (K.B.); edkitcher@hotmail.com (E.K.); 3 School of Life Sciences, University of Sussex, Brighton BN1 9QG, UK; ew323@sussex.ac.uk

**Keywords:** nasopharyngeal cancer, Epstein Barr virus, genotypes, viral load, Ghana

## Abstract

Nasopharyngeal cancer (NPC) is associated with Epstein Barr virus (EBV) infection. However different viral strains have been implicated in NPC worldwide. This study aimed to detect and characterize EBV in patients diagnosed with NPC in Ghana. A total of 55 patients diagnosed with NPC by CT scan and endoscopy were age-matched with 53 controls without a known oncological disease. Venous blood was collected from the study participants and DNA extracted from the blood samples. Detection of EBV and genotyping were done by amplifying Epstein Barr nuclear antigen 1 (EBNA-1) and Epstein Barr nuclear antigen 2 (EBNA-2), respectively, using specific primers. Viral load in patients and controls was determined using real-time polymerase chain reaction. EBV positivity in controls (92%) was significantly greater than that of NPC patients (67%) (χ2 = 19.17, *p* < 0.0001), and viral infection was independent of gender (χ2 = 1.770, *p* = 0.1834). The predominant EBV genotypes in patients and controls were genotype 2 (52%) and genotype 1 (62%), respectively. Median EBV load was significantly higher in NPC patients than the control group (*p* < 0.01). In summary, prevalence of EBV genotype 2 infection was higher in NPC patients than the control group. Assessment of EBV load may be used as a biomarker for the diagnosis of NPC.

## 1. Introduction

Nasopharyngeal cancer (NPC) is a malignant tumor of epithelial squamous cell which arises from the lateral wall of the nasopharynx, notably, the fossa of Rosenmüller located superior and posterior to the torus tubarius [1,2]. NPC has an incidence of 1 person per 100,000 in non-endemic regions, with an incidence of 25 to 30 persons per 100,000 in endemic regions like China and Southeast Asia [3]. In Africa, specifically Northern Africa, the yearly incidence of NPC varies between 5 to 7 persons per 100,000 [4]. NPC is one of the leading cancers in Ghana, with a prevalence of 29% among the head and neck cancers [5], and about 1.2% of all cancers [6]. There are ethnic and geographic variations in the occurrence of NPC, which indicates a multifactorial etiology involving EBV infection, genetic susceptibility, ethnicity, environmental factors and food consumption [7,8].

The most common etiological factor associated with NPC development is Epstein Barr virus (EBV) infection. EBV is a ubiquitous oncogenic virus belonging to the family Herpesviridae [9], which is further classified into subfamilies: alpha-, beta-, and gammaherpes virus. EBV is a classical member of the subfamily Gammaherpesvirinae, and among nine viruses of the subfamily that have been identified to solely infect humans [10,11]. Two genotypes of the virus, namely genotypes 1 and 2, exist and exhibit variation in geographical distribution. Although EBV genotype 1 is globally distributed, it is predominantly found in American, Chinese, European and South-East Asian (SEA) populations, whereas genotype 2 is predominantly found in Africa [12]. The two genotypes also vary in biological properties; EBV genotype 1 is more efficient in immortalizing B cells while genotype 2 has a higher lytic ability [13,14].

Despite the increased number of NPC cases reported globally, little has been done to characterize EBV and determine the association of EBV genotype with NPCs, especially in Africa. The current case-control study was aimed at determining the prevalence of EBV infection and the viral genotype associated with NPC in Ghanaian patients who are not undergoing cancer therapy.

## 2. Methods 

### 2.1. Study Site

This study was conducted at the Ear, Nose, and Throat (ENT) Unit of the Korle-Bu Teaching Hospital (KBTH), Accra. The Unit serves as a referral center for nasopharyngeal cancer (NPC) cases in Ghana and the West African region. 

### 2.2. Recruitment of Study Participants 

A total of 108 study participants reporting to the ENT Unit of the KBTH from April 2018 to March 2019 were enrolled into the study. Out of the total number of recruited participants, 55 were suspected cases of NPC, which was determined by endoscopy and computed tomography (CT) scan, although biopsies from the participants were yet to undergo histopathological examination. The remaining 53 participants were NPC negative subjects and served as controls. A well-structured questionnaire was administered to acquire demographic data from the study participants after informed consent had been obtained. The study was approved by the Institutional Review Board of the Noguchi Memorial Institute for Medical Research (NMIMR) with reference number 051/16-17. 

### 2.3. Collection of Blood Samples and DNA Extraction 

Venous blood (5 mL) was collected from the study participants and transferred into EDTA tubes. Genomic DNA was extracted from the blood samples using Qiagen QIAamp Blood Mini Kit (QIAGEN, Hilden, Germany) by following the manufacturer’s protocol. The eluted DNA was stored at −20 °C until ready to use.

### 2.4. Positive Controls

Plasmids pJET-EBNA-1 and pJET-EBNA-2 containing EBV DNA were used as positive controls for EBV detection, and EBV genotypes 1 and 2, respectively. Moreover, pJET-Beta plasmid was used as positive control for beta globulin. 

### 2.5. Detection of EBV by Amplification of Epstein Barr Nuclear Antigen-1 (EBNA-1)

The extracted genomic DNA samples were screened for the presence of EBV by polymerase chain reaction (PCR) amplification of EBNA-1 using primers (QP1, QP2) that have previously been reported, and for the human beta globulin gene as internal control (Table 1). Each PCR reaction mixture of 12.5 µL contains the following components with the final concentrations: 1X One TaqR Quick-Load 2X Master Mix with Standard Buffer (New England Bio Labs, Hertfordshire, UK), 0.5 µM each of the forward and reverse primers, and 0.5 µg/µL of genomic DNA. The volume was made up with nuclease-free water. PCR reaction cycling conditions involved initial denaturation at 94 °C for 30 s, followed by 35 cycles of amplification with denaturation at 94 °C for 30 s, annealing at 56 °C for 1 min, extension at 68 °C for 1 min, and a final extension at 68 °C for 5 min. An aliquot of 8 μL of the amplicons were resolved on 2% ethidium bromide-stained agarose gel, and bands were visualized using Amersham gel imager.

### 2.6. EBV Genotyping by Epstein Barr Nuclear Antigen 2 (EBNA-2) Nested PCR Amplification 

The genotypes of EBV in both patients and controls were detected by nested PCR using specific primers as previously reported (Table 1), with slight modifications to cycling conditions. The first round of the PCR was done by amplifying a common region of EBNA-2 using EBNA-2F and EBNA-2I as sense and antisense primers, respectively. Each PCR reaction mixture of 12.5 µL contains the following components with the final concentrations: 1X One TaqR Quick-Load 2X Master Mix with Standard Buffer (New England Bio Labs, UK), 0.5 µM each of the forward and reverse primers, and 0.5 µg/µL of genomic DNA. The cycling conditions for the reaction were as follow: initial denaturation at 94 °C for 2 min, followed by 35 cycles of amplification with denaturation at 94 °C for 60 s, annealing at 52 °C for 90 s, extension at 72 °C for 4 min, and a final extension at 72 °C for 10 min. A second round PCR (nested) was performed using 0.5 μL of the amplicons from the first round as template; all other reaction components were the same as the first-round reaction mixture, except for the primers. A forward primer (EBNA-2C), that is common to both genotypes, and reverse primers EBNA-2G and EBNA-2B, which are specific for the respective genotypes were used for the second-round amplification. The reaction was carried out at initial denaturation of 94 °C for 2 min, followed by 35 cycles of amplification with denaturation at 94 °C for 30 s, annealing at 52 °C for 60 s, extension at 72 °C for 2 min and a final extension at 72 °C for 10 min. Eight microliters of the amplicons were resolved on 2% ethidium bromide-stained agarose gel and visualized using the Amersham gel imager.

### 2.7. EBV Load Quantification in Whole Blood by EBNA-1 Real-Time PCR

Measurement of EBV load in blood samples was carried out by real-time PCR, QauntStudio5 PCR System (Applied Biosystems, Waltham, MA, USA). The genomic DNA samples were used for the quantification of EBV by amplifying EBNA-1 and beta-globulin gene (internal control). The PCR reaction volume was made up of the following components: 5 μL 1X Sybr Green SuperMix (Quantabio, Beverly, MA, USA), 0.5 µg/µL template DNA, 0.1 μL of 0.1 μM each of primers (QP1, QP2, B-globulin forward and reverse) (Table 1), and 3.8 μL of nuclease free water. The cycling conditions consisted of a hold at 95 °C for 10 min, followed by an initial denaturation step at 95 °C for 10 min, and then 40 cycles of denaturation at 95 °C for 15 s and 60 °C for 1 min re-annealing and extension. All samples were analyzed in duplicates. A standard reference curve was obtained by amplifying a 10-fold serial dilution of EBNA-1 DNA in pJET-EBNA-1 plasmid. EBV load was expressed as log EBV DNA copies/mL. 

### 2.8. Statistical Analysis 

Statistical analysis was performed using IBM SPSS statistical software version 20 and GraphPad Prism 8. Categorical variables were compared using Chi-squared test of independence for contingency table and corrected by Yates’ continuity test or Fisher’s Exact test. After confirmation of the normal distribution of EBV load results by Kolmogorov and Smirnov tests, Mann–Whitney rank-sum test was used to compare median viral load between cases and controls for binary categories. A *p*-value less than 0.05 was considered statistically significant. 

## 3. Results

### 3.1. Demographics of Study Participants and Molecular Detection of EBV in Whole Blood

**Figure 1:**
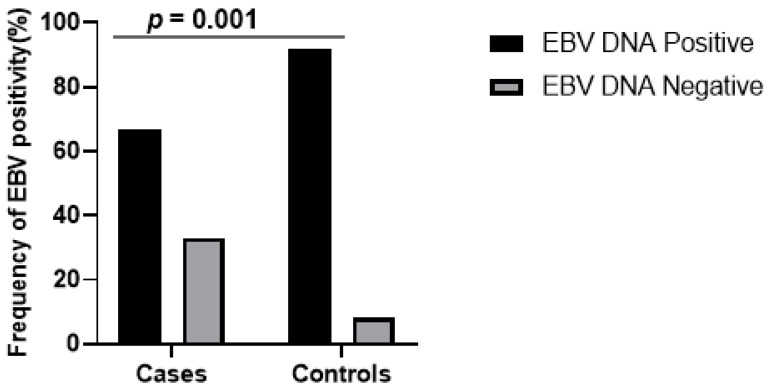
Graph showing frequency of EBV positivity in NPC cases and controls. Frequency of EBV positivity was significantly higher in control subjects than cases, when comparison was done using Chi-squared test of independence for contingency table (*p* = 0.001).

The median ages of the patients and controls were 40 and 47 years, respectively. The proportion of males suspected with NPC and those in the control group were 47% (26/55) and 30% (16/53), respectively. Furthermore, the proportion of females suspected with NPC and those in the control group were 53% (29/55) and 70% (37/53), respectively. EBV DNA was detected by EBNA-1 amplification (213 bp) (Figure S1) and the number of positives were 67% (37/55) and 92% (48/53) for patients and controls, respectively. The EBV DNA positivity was significantly higher in controls than the patients (χ2 = 19.17, *p* < 0.0001) (Figure 1). The EBV DNA positivity in females (81.8%) was significantly higher than in males (66.7%). If we assume EBV DNA positivity represents EBV infection history, it means that females were two times more at risk of having EBV infection than males (OR = 2.24, 95% CI = 1.16–4.34, *p* = 0.0225).

### 3.2. EBV Genotypes in NPC Patients and Controls of the Study Participants

**Figure 2:**
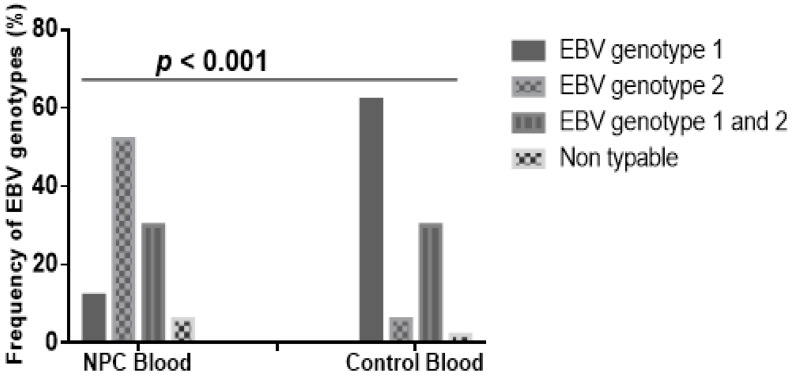
Frequency distribution of EBV genotypes in cases and control samples. Frequency of EBV genotype 1 was significantly higher in control subjects than cases, whereas frequency of EBV genotype 2 was significantly higher in cases than controls, when comparison was done using Chi-squared test of independence for contingency table (*p* < 0.001).

EBV genotyping using the EBNA-2 gene showed 250 bp and 300 bp fragments which are characteristic of genotypes 1 and 2, respectively (Figure S2). Frequencies of EBV genotype 1 in blood samples of NPC patients and controls were 11.5% (6/52) and 62.3% (33/53), respectively, whilst genotype 2 was 51.9% (27/52) in the NPC patients compared to 5.7% (3/53) in controls (Figure 2). Co-infection with EBV genotypes 1 and 2 in NPC cases was 30.8% (16/52) and in 30.2% (16/53) in the controls. Differences in EBV genotype distribution in both patients and controls were statistically significant (*p* < 0.001); the EBV genotype 1 was higher in the controls than the patients (χ2 = 51.5, *p* < 0.001), and the reverse was observed for EBV genotype 2 (χ2 = 49.2, *p* < 0.001). Individuals infected with EBV genotype 2 were almost 17 times more at risk of developing NPC (OR = 16.97, 95% CI = 6.86–42.32, *p* < 0.0001) compared to the weakly associated genotype 1 (OR = 0.08, 95% CI = 0.04–0.17).

### 3.3. EBV Load in NPC Patients and Controls

**Figure 3:**
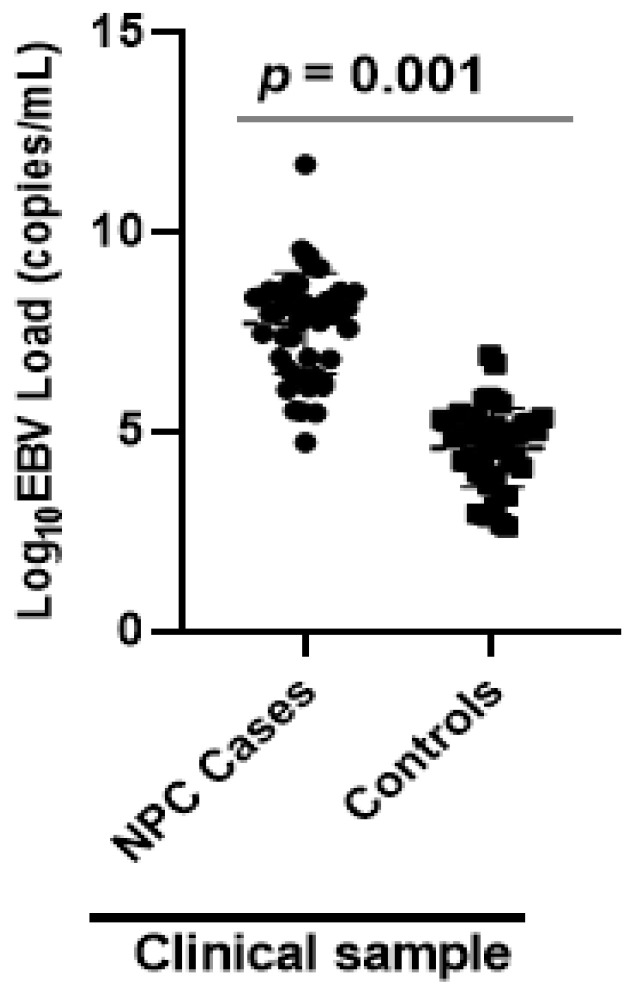
Comparison of EBV load (copies/mL) in whole blood samples of NPC patients and control group. Epstein Barr virus (EBV) load quantification in whole blood samples was done by EBNA-1 real-time PCR in nasopharyngeal cancer (NPC) patients and healthy controls. EBV copies are represented on logarithmic scale (Log_10_). Comparison of the median EBV load between cases (*n* = 37) and controls (*n* = 48) was performed using a Mann–Whitney test. EBV load was significantly higher in NPC patients than controls (*p* = 0.001).

The median Epstein Barr viral load of 9 × 10^7^ copies/mL (53 × 10^3^ to 5 × 10^11^ copies/mL) in NPC patients was significantly elevated when compared to controls of 6 × 10^4^ copies/ mL (4 × 10^2^–8 × 10^6^ copies/mL) (*p* < 0.01) (Figure 3). 

## 4. Discussion 

Nasopharyngeal cancer (NPC) is a malignant tumor of epithelial squamous cells which arises from the lateral wall of the nasopharynx, notably, the fossa of Rosenmüller, which is located superior and posterior to the torus tubarius [2]. The etiology of NPC is commonly associated with EBV infection. Although EBV infects more than 90% of the world’s population, most of the infections are asymptomatic [3]. The replication of the virus is held in check by EBV-specific cytotoxic T cells in immunocompetent individuals. However, in instances where immune surveillance by cytotoxic T cells wanes, the viral outgrowth is enabled, and this may result in various EBV-associated cancers [15]. In the host, EBV expresses restricted sets of latent genes that vary from one malignancy to another, as well as in asymptomatic carriers [16]. 

In the current study, we detected EBV EBNA-1 gene in the whole blood of both patients suspected of NPC and control subjects without a known oncological disease. The virus was present in over 90% of the control group of the study and supported the finding in literature that about 90% of the world’s population are asymptomatic carriers of the virus [17]. A public health factor that may contribute to high prevalence of EBV in healthy individuals is the infection risk of EBV transfer through blood transfusion, and this has been reported in some studies [18,19]. However, the results obtained from the current study, which suggest a high prevalence in asymptomatic carriers, was inconsistent with results reported from similar studies that have been conducted in other parts of the world [3,20]. With regards to females being two times more at risk of having EBV infection compared to males, the authors suspect this observation could be due to hormonal differences. There was, however, no information in the literature to explain the suspicion, and therefore the observation remains a subject for further investigation. 

EBV genotype 2 was the predominant genotype in NPC patients whilst the genotype 1 was common in the controls. This observation was made from the genotyping of EBV in the NPC cases and control samples, and consistent with the genotype 2 being the virulent type. To the best of our knowledge, this study is the first and provides a baseline information on the prevalence and co-infection patterns of the genotypes in Ghanaian patients. Our research group is currently investigating the pathogenicity of the two EBV genotypes (type 1 and type 2). The follow-up study will associate virulent factors with the EBV genotypes, and possibly suggest a mechanistic role of the factors in disease etiology, which will ultimately drive the search for therapeutic targets. The classification of EBV into the two main geographically distinct genotypes, 1 and 2, is based on sequence polymorphism in EBNA-2, -3A, -3B and -3C [21,22]. However, since EBNA-2 has the least percentage of sequence homology EBV typing has been done using this protein [17]. EBV genotype 1 has been reported as the predominant genotype globally, and both NPC patients and healthy individuals showed similar viral distribution of the genotype [14,23]. Studies have mapped geographical regions with high prevalence of NPC and EBV genotypes. Moreover, EBV genotypes 1 and 2 co-infected in both NPC patients and healthy participants, which is consistent with reports from other parts of the world [14,24]. 

The study patients showed significantly high EBV load than the control group. This conclusion was drawn from the quantitative PCR amplification of the EBNA-1 gene, which had high levels of viral DNA compared to the controls. A previous report showed that, NPC patients with significantly higher EBV DNA level developed local recurrence or distant metastasis with high risk of disease-associated mortality within a year of treatment [25]. Quantification of EBV load in NPC patients has proven useful in prognostication, early detection, tumor staging and monitoring of treatment response [26]. Many studies have quantified EBV load in plasma, serum and saliva samples from NPC patients [25,27,28,29], but only a few studies have been done in whole blood specimens [30,31]. The current study therefore presents for the first-time whole blood EBV load in Ghanaian patients suspected of NPC and naïve to chemotherapy. The high viral load observed in whole blood from NPC patients was consistent with findings from other parts of the world [32,33]. EBV establishes latent infection in memory B cells at a low viral level after primary infection, and hence, healthy individuals may carry quantifiable EBV loads in blood circulation [34]. In NPC patients, however, whole blood EBV load is derived from cancerous epithelia tumor cells and circulating B cells. The DNA from the epithelial and B cells may account for the significantly high viral load in patients compared to healthy controls [35]. 

Despite the above findings, the results from the current study should be explained with the consideration of some limitations. The limitations to this study are the relatively small sample size used, and the unavailability of histopathological results to confirm the NPC status of the patients. 

This study identified EBV genotype 2 infection as the virulent genotype in Ghana and a possible risk factor in the development of NPC in Ghanaian patients. EBV genotype 1 was predominant in the control group and consistent with the genotype being relatively less virulent. The detection and quantification of EBV load can be used as a non-invasive biomarker for the diagnosis of NPC.

## Figures and Tables

**Table 1:** Primers used for EBV detection, genotyping and beta globulin amplification.

Gene Target	Primer Name	Primer Sequence	Amplicon Size
EBNA-1	QP1 (forward)	(GCC GGT GTG TTC GTA TAT GG)	213 bp
	QP2 (reverse)	(CAA AAC CTC AGC AAA TATATG AG)	
EBNA-2	**First round primers**		801 bp
	EBNA-2F (sense)	(TGGAAACCCGTCACTCTC)	
	EBNA-2I (antisense)	(TAATGGCATAGGTGGAATG)	
	**Second round primers**		250 bp 300 bp
	EBNA-2C (common sense primer)	(AGGGATGCCTGGACACAAGA)	
	EBNA-2G (type-1 antisense)	(GCCTCGGTTGTGACAGAG)	
	EBNA-2B (type-2 antisense)	(TTGAAGAGTATGTCCTAAGG)	
Beta globulin	B-globulin F	(ACACAACTGTGTTCACTAGC)	119 bp
	B-globulin R	(CAACTTCATCCACGTTCACC)	

## Supplementary Materials

The following are available online at https://www.mdpi.com/1999-4915/12/7/766/s1, Figure S1: Gel electropherogram of EBV EBNA-1 PCR amplification, Figure S2: Gel electropherogram of EBV genotyping.
