# Supplementary figures and images for: Genotypic Characterization of Epstein Barr Virus in Blood of Patients with Suspected Nasopharyngeal Carcinoma in Ghana
Source: Viruses. 2020 Jul 16;12(7):766. doi: 10.3390/v12070766 (PMC7412455; doi:10.3390/v12070766)

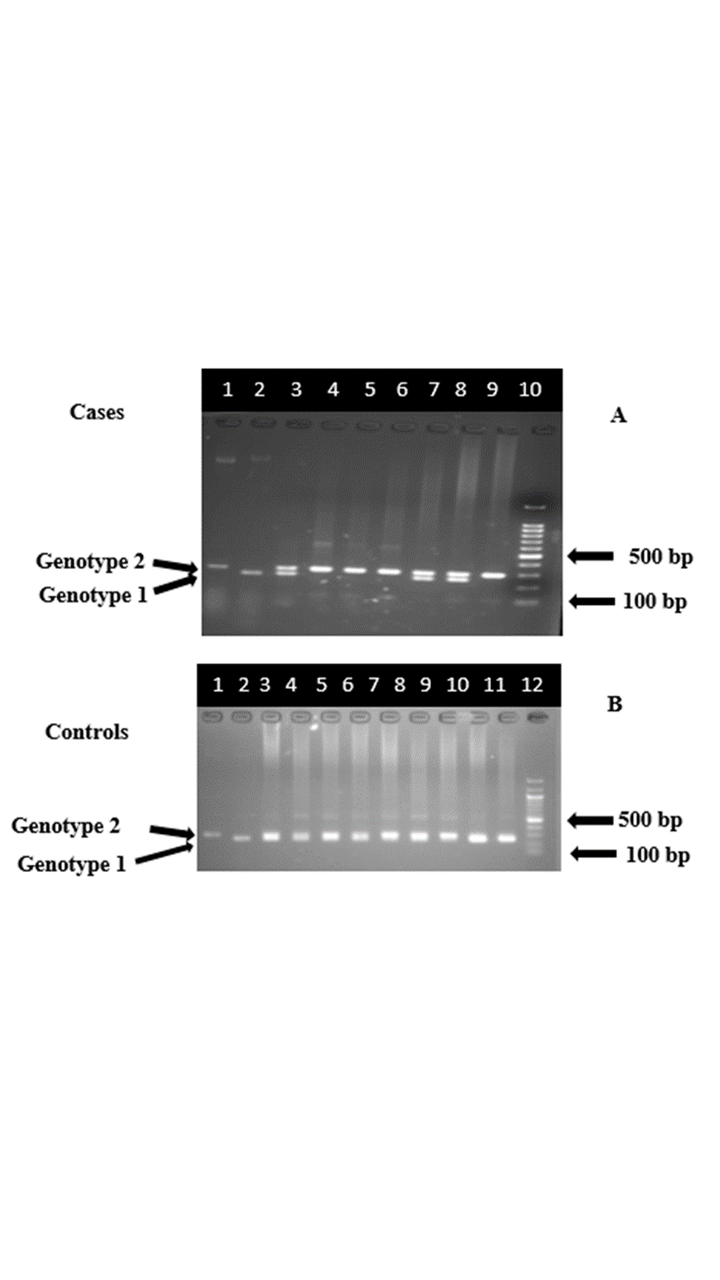

Supplement: Supplementary file 1 [file viruses-12-00766-s001.zip › viruses-806775-suppl/Supplementary Figure 1.tif]

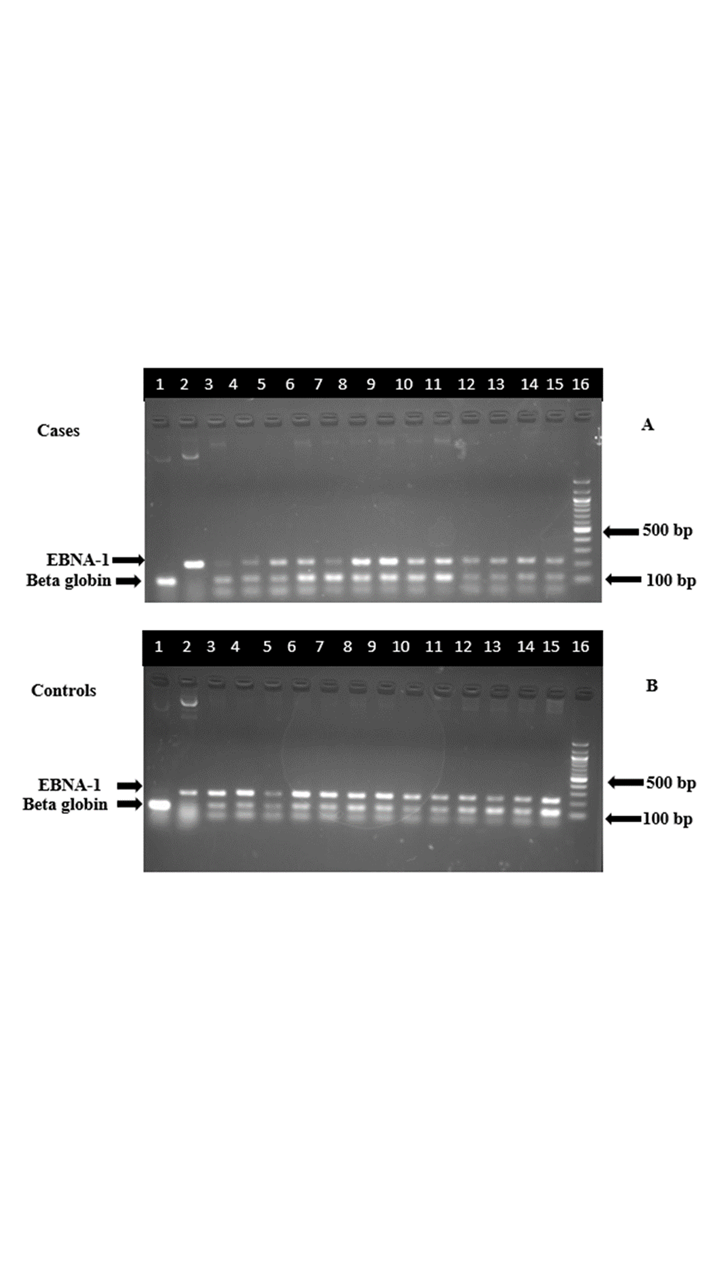

Supplement: Supplementary file 1 [file viruses-12-00766-s001.zip › viruses-806775-suppl/Supplementary Figure 2.tif]
